# The development and validation of the hospital organizational environment scale for medical staff in China

**DOI:** 10.3389/fpubh.2023.1118337

**Published:** 2023-09-21

**Authors:** Yu Wang, Jingwen Zhang, Xingmiao Feng, Yan Liang, Zhongjun Guan, Kai Meng

**Affiliations:** ^1^School of Public Health, Capital Medical University, Beijing, China; ^2^Shunyi Hospital of Beijing Traditional Chinese Medicine Hospital, Beijing, China; ^3^Beijing Tiantan Hospital, Capital Medical University, Beijing, China

**Keywords:** medical staff, questionnaire, development, validation, organizational environment

## Abstract

**Objectives:**

There is currently no measure of the hospital organizational environment targeting both clinicians and nurses in China. This study was conducted with the aim of developing and testing an instrument to assess the properties of the hospital organizational environment that is applicable to Chinese medical staff.

**Methods:**

Items were developed based on a literature review, semi-structured interviews and an expert review and finalized based on corrected item-total correlation, content validity, construct validity, convergent validity, discriminant validity and reliability. The two samples for testing the first and final version of the Hospital Organizational Environment Scale (HOES) included 447 and 424 participants, respectively.

**Results:**

The primary test, which comprised 18 items, contained four factors: hospital culture, work situation, organizational support and scientific research situation. The Cronbach’s alphas were 0.935, 0.824, 0.943, and 0.920, respectively. The results of the validation test showed that the questionnaire had good validity and reliability.

**Conclusion:**

The HOES is a comprehensive instrument with demonstrated validity and reliability that can be adopted among medical staff to assess the organizational environment in hospitals.

## Background

The “organizational environment,” also called the “work environment,” is studied in environmental psychology and can be divided into two types: the physical environment and social environment (1). Early studies of environmental psychology mostly focused on the impact of the physical environment on people’s mental health and behavior, including factors such as noise, air pollution, climate, and related architectural design (1–3). With the change in social problems, environmental psychology focuses more on exploring the relationship between social environment factors and human behavior (4, 5). Hence, in the study, the organizational environment comprised the psychological and social environment perceived by employees.

There is still a lack of consensus about how the hospital organizational environment is best conceptualized, which directly affects the different scales and dimensions used in the assessment of the organizational environment. The “organizational environment” affects organizational goal setting and operation behaviors, and it can further influence organizational task performance according to Dill (6). Claire Capon’s view (7) that the organizational environment mainly includes organizational culture, organizational resources and functions, and member behavior. Aiken (8) believed that the work environment of a hospital can be understood as the internal environment of the organization, which is affected by the work situation, doctor–patient relationships, the organizational culture, etc. The American Association of Critical-Care Nurses (AACN) (9) suggested that the hospital work environment needs to provide organizational support to meet the autonomy needs, the positioning of values and management methods at the organizational system level. In literature reviews (4, 5, 7, 10, 11), numerous theories and different definitions of the work environment have been identified due to differences in research objectives and fields. Based on the above studies, the key elements of the organizational environment include the following four: the hospital organizational culture (12), referring to the cultural mentality, ideology and behavior norms formed by medical personnel in medical practice. And organizational support (13), considering as the overall perception that the organization values their own contribution and pays attention to their well-being. Doctor–patient relationships (14), referring to medical staff’s perception of the relationships with patient’s in the process of clinical practice. Work situation (15), referring to perception of workload or work-related factors. It can be concluded that the hospital organizational environment is a multidimensional concept referring to the sum of various psychosocial elements of the management system and organizational atmosphere that directly or indirectly affect the mental health and behaviors of medical staff; the hospital organization environment can further influence organizational goal setting and task performance.

The existence and development of any organization is inseparable from its environment, which enables information exchange and resource sharing. If the organizational environment is inconsistent with people’s needs, it will have negative effects, such as stress and dissatisfaction (11). For example, due to the special organizational environment of hospitals, health care professionals come into contact with serious diseases and death every day and experience greater physical and psychological pressure than individuals in other professions (16, 17). Aiken et al. (18) found a negative correlation between the work environment and burnout. In addition, Chan and Huak (19) found that a high proportion of doctors and nurses suffered from mental disorders, anxiety, depression and posttraumatic stress disorder. A harmonious hospital work environment can not only reduce the levels of burnout and promote the mental health of medical staff (20) but also improve the quality of medical services for patients (21). Thus, the hospital organizational environment is very important to promote the physical and mental health of medical staff.

Evaluating the hospital organizational environment can help determine medical staff members’ feelings about the hospital and strengthen hospital management. The scope and structure of the assessment of the hospital organizational environment should be clearly defined according to the change in the actual situation and cultural context. However, limitations regarding the current hospital organizational environment have been identified in studies, and most of the measurements of the organizational environment were designed based on psychological scales combined with nursing characteristics (20–23). To date, the most widely used organizational environment scale is the Nursing Work Evaluation Index (Nursing Work Index-Revised) (24–26). It was primarily constructed from the perspective of nursing work practice and not from the perspective of the entire organizational system.

Some applicable conditions need to be considered. First, the hierarchical medical system in China is not perfect (27), and tertiary public hospitals undertake most of the medical treatment work. Chinese doctors are not allowed to engage in private practice, so patient disease management needs to be considered in the clinical practice of both nurses and doctors together. Thus, nurses and doctors are confronted with a similar organizational environment and the same clinical workload. In addition, public hospitals employ performance management measures that combine the personal goals of the medical staff with organizational strategic goals. The ability to conduct scientific research is incorporated into the performance appraisal system (28). For Chinese medical staff, promotion to a professional title requires not only excellent clinical practice skills but also certain scientific research abilities. Moreover, scientific research abilities and achievements are also indispensable factors for medical staff in hospital performance evaluations. Chinese medical staff experience serious pressure to perform scientific research in the current organizational environment. There is currently no measurement of the hospital organizational environment that targets both clinicians and nurses who face similar work circumstances. Additionally, current measurement methods fail to take into account both the scientific research stress and clinical workload that nurses and doctors are commonly confronted with. Hence, a universally applicable instrument needs to be developed. Therefore, this study was conducted with the aim of developing and testing an instrument to assess the properties of the hospital organizational environment that is applicable to Chinese medical staff.

Therefore, in this research, the widely recognized concept was summarized in four dimensions (hospital organizational culture, organizational support, doctor–patient relationships, working situation) to determine the basic structure of the hospital organizational environment. Then, in the development phase, we conducted qualitative interviews to collect and discover more information to expand the boundaries of the hospital organizational environment dimension. Finally, we developed a universally applicable instrument assessing the organizational environments of Chinese hospitals.

## Methods

### Study design overview

**Figure 1:**
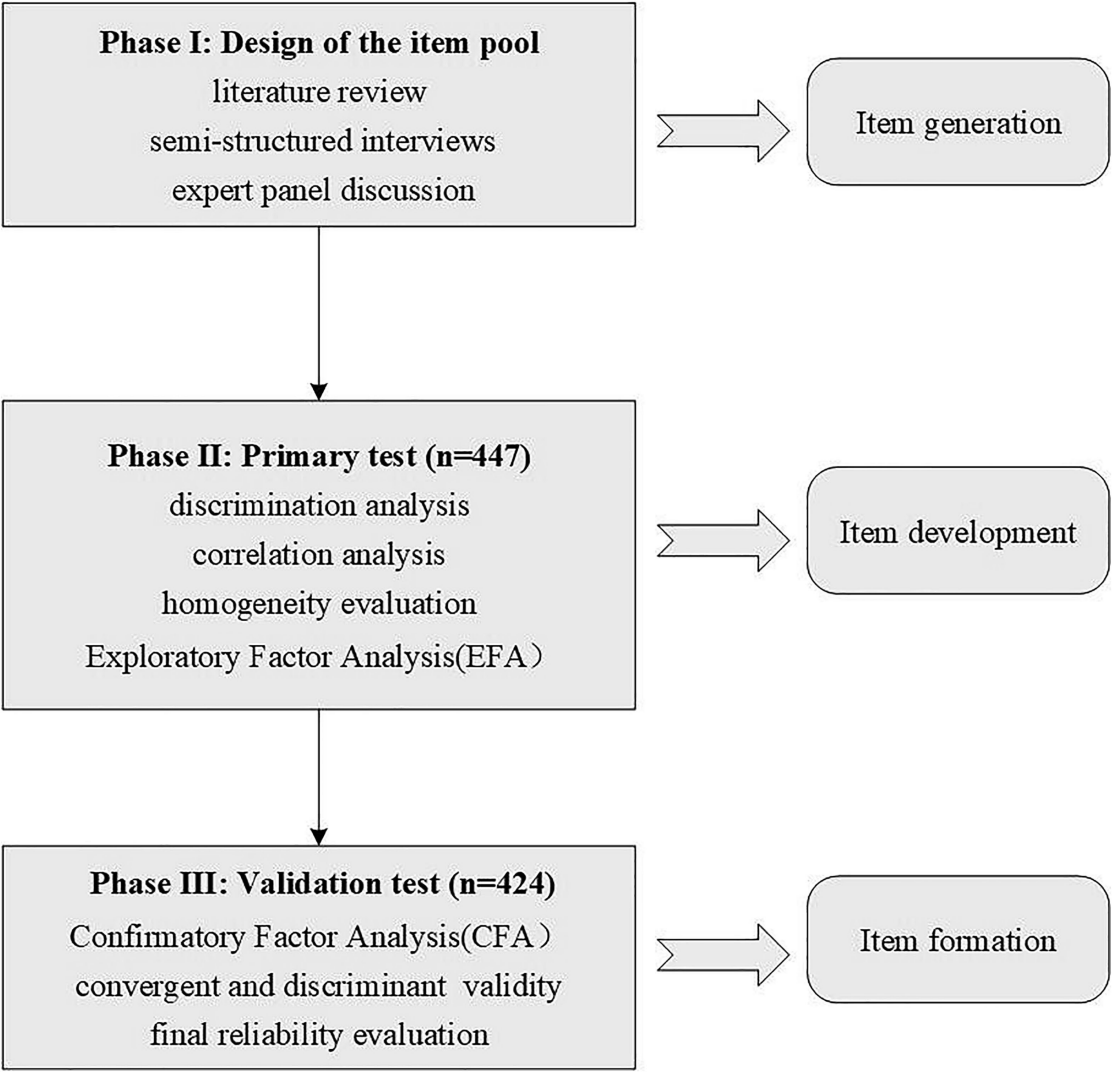
Scale construction process.

The study was performed in three stages: In Phase I, The Hospital Organizational Environment Scale (HOES) items were generated through a comprehensive literature review, semi-structured interviews and discussion with an expert panel specializing in health service management. The experts’ opinions regarding the wording, language, ease of use and generalizability to practice were incorporated into the instrument. The content validity was analyzed based on the experts’ opinions. Assessments of construct validity included an exploratory factor analysis (EFA) and a confirmatory factor analysis (CFA). In Phase II, the primary test was developed through EFA to ensure that the items were readable, with no lack of clarity or reliability. Then, in Phase III, a validation test was performed through CFA and convergent and discriminant validity analysis to ensure that the scale was valid, explicit and accurate in reflecting the organizational environment among medical staff. The scale construction process is shown in Figure 1.

### Patient and public involvement statement

Neither patients nor the public were involved in this study, as this research focused solely on scale development.

### Phase I: design of the item pool

#### Literature research method

The literature research method (29) mainly included two parts: the first part involved determining the theoretical structure of the public hospital organizational environment scale for medical staff in China; the second part involved determining the specific content of the scale for medical staff in China.

The data sources included the following electronic databases: Google Scholar, PubMed, Web of Science, MEDLINE, CNKI, WanFang and CBM. To review the literature, we used the following search terms: (Tertiary public hospitals [Title] + (hospital[Title])*(organizational environment [Title] + (work environment [Title])*(organizational support [Title]) + (organizational culture [Title]) + (doctor–patient relationships [Title]) + (work situation)[Title] + (work stress)[Title])*((evaluation[Title]) + (assessment[Title]) and ((organizational environment [Title] + (work environment [Title])*((organizational support [Title]) + (organizational culture [Title]) + (doctor–patient relationships [Title]) + (work situation)[Title] + (work stress)[Title])*(evaluation [Title]) + (assessment[Title] + (scale [Title/Abstract]). The inclusion criteria consisted of all indicators of the hospital organizational environment, including the hospital culture, working situation, organizational support and doctor–patient relationships. The exclusion criteria were indicators that could not be applied to evaluate the organizational environment or indicators with repeated formulations or descriptions. After duplicates and conference reports were removed, 24 papers remained, and based on the team members’ intensive reading, 8 papers were considered for inclusion. The retrieved articles (5, 12–15, 24–26) were assessed independently by two authors, information was extracted from the eligible studies, and the English items were preliminarily translated.

#### Semi-structured interviews

In order to clarify and develop the framework of the theoretical dimensions of the organizational environment, we conducted semi-structured interviews to gain a deeper understanding of the connotation of the hospital organizational environment (30). Twenty medical staff were randomly selected for face-to-face semi-structured interviews that served as a supplement to the current Chinese hospital organizational environment dimensions. All interviewees agreed to the whole process being recorded. The interviews were conducted by three graduate students from the Department of Public Health, Capital Medical University. The interview question was “What do you think the elements of the current organizational environment in public hospitals are?” At the same time, the interviewees’ answers were recorded, and when the answers did not involve the content of the mainstream scale, the interviewer asked whether the factors in this aspect were related to the organizational environment until enough interview data were collected to achieve information saturation. Then, members of the research group analyzed the interview results and two experienced bilingual medical experts checked and revised the translated items.

#### Content validity

Content validity refers to the extent to which the content of a scale reflects or represents the construct that a researcher intends to measure (31). In current practice, qualitative methods are used to evaluate the content validity of a scale. Content validity was assessed based on the following criteria: appropriateness, comprehensibility and clarity of phrasing for all items. The expression of each topic should be as concise and clear as possible, be easy to understand, and have wording. In this study, we invited experts in the field of health service management to make independent judgments based on their own knowledge and work experience, assess the content and expression of each topic, and delete or revise inappropriate or inaccurate topics in all the originally prepared topics. In this study, each item was scored on a five-point Likert scale ranging from “1 = very strongly disagree” to “5 = very strongly agree” (1–5), and seven items were reverse scored.

### Phase II: primary test

This stage involved item reduction and the development of the primary test. Sufficient quantity and standard-compliant participants were selected for the EFA to extract key components.

#### Sample and setting

It is generally accepted by most researchers in the field of social and behavioral sciences that results are more reliable than pretest samples based on the number of items (32). According to the literature report, when evaluating the properties of a scale, the testing sample size should be 5–10 times the number of analysis items (33). In our study, the number of participants in each stage met this condition. Cluster sampling was adopted in this study, and all medical staff were recruited from Hospital Y. There were 416 practicing physicians and 600 registered nurses in Hospital Y. There were 750 hospital beds in Hospital Y (a tertiary hospital should have at least 500 beds). The participants had to meet the following criteria: (1) were registered clinicians or nurses with at least 1 year of clinical or nursing practice experience and (2) agreed to voluntarily participate in this project and signed the informed consent form. Those who were unwilling to cooperate during the investigation were excluded. The questionnaire was developed in the Chinese language. The questionnaire collected demographic information (e.g., age, sex, marital status, professional title, and the number of years of medical work experience) and contained 22 initial HOES items scored on a 5-point scale from 1 (strongly disagree) to 5 (strongly agree).

#### Descriptive analysis

Floor and ceiling effects are considered to be present if more than 15% of respondents achieve the lowest or highest possible score, respectively (34). Skewness and kurtosis are rough indicators of a normal distribution of values: skewness is an index of the symmetry of a distribution, while kurtosis is a measure used to describe the tailedness (35). Symmetric distributions have a skewness value of 0 and a kurtosis value of 3 (36). If the skewness value is less than 3 and the kurtosis value is less than 10 (37), it is regarded as basically acceptable that the sample obeys a normal distribution.

#### Discriminant analysis

The discrimination value refers to the difference between the percentage of correct answers in the high group (the first 27% of the subjects) and that in the low group (the last 27% of the subjects) (38). The main purpose of analyzing the discrimination index value was to determine whether the test could distinguish subjects’ abilities. The average score of each item was compared between the high and low groups. We adopted the independent sample *t* test to assess the differences between participants in the high and low groups. The *t* value obtained was called the critical ratio (CR), and *p* < 0.05 indicated the significance of the items.

#### Correlation analysis

This method filters items from the perspective of representativeness and independence (39). We adopted the Pearson correlation coefficient to measure the correlations. The least relevant item was excluded due to its high theoretical association with the same underlying dimension. In this study, the score correlation coefficient between each item and the total items was statistically calculated. A coefficient greater than 0.4 (40) indicated that each item had good representativeness in its dimension.

#### Homogeneity evaluation

If the standardized Cronbach’s α coefficient of a scale increases after a variable is deleted compared with that before deletion, it indicates that the variable has a hidden danger of reducing the internal consistency of the scale and that the corresponding items should be considered for deletion (38).

#### Exploratory factor analysis

EFA extracts a certain number of common factors from all items according to the structure envisaged by the measuring tool and considers the composition of each principal component according to the results of common factor extraction and the load of each index on the common factor. In this study, first, the suitability condition of the EFA was assessed by Bartlett’s test (41) and the Kaiser–Meyer–Olkin (KMO) measure. According to Kaiser (42), whether items are suitable for factor analysis can be judged from the KMO index value. A KMO sampling adequacy value greater than 0.90 indicates that the relationship between item variables is excellent (43). Then, factors with eigenvalues >1 were retained (44). Factor analysis with the maximum variance method was used to extract the principal factors of the organizational environment. Then, the factor loading matrix was obtained by the Kaiser standardized orthogonal rotation method. The loading of the item on the principal factor was required to be greater than 0.50. If the loading value for the item on each principal factor was less than 0.5, deletion was considered when the loading values on two or more principal factors were greater than 0.5 (45).

### Phase III: validation test

In this stage, a validation test was performed for item formation, and eligible participants were selected to perform the CFA using M-Plus 8.0.

#### Sample and setting

Using the same inclusion and exclusion criteria as above, study participants were enrolled from Hospital S. There were 403 practicing physicians and 402 registered nurses in Hospital S, which had 450 hospital beds. Confirmatory analysis was performed for Hospital S. The questionnaire collected demographic information (e.g., age, sex, marital status, professional title, and the number of years of medical work experience) and contained 18 initial HOES items.

#### Convergent and discriminant validity

The convergent and discriminant validity of the instrument were evaluated through Fornell and Larcker’s (46) approach using the average variance extracted (AVE) and composite reliability (CR). Convergent validity is confirmed if the items of the intended scale show strong correlations. In addition, discriminant validity is supported when the extracted factors are distinct from each other. To confirm convergent validity, the AVE should be greater than 0.5, and the CR value should be greater than the AVE. However, discriminant validity is maintained if the AVE is greater than the maximum shared squared variance (MSV) and the average of squared variance (ASV).

#### CFA

We performed a CFA (47) to test the fitness of the factor structure extracted from the original 4-factor subscales of the 18-item scale. The extracted factor model was evaluated via maximum likelihood estimation using the following model fit indices (48, 49): the comparative fit index (CFI), Tucker–Lewis index (TLI), root mean score error of approximation (RMSEA), freedom (CMIN/DF), standardized root mean square residual (SRMR), chi-square test of model fit and degrees of freedom (*χ*^2^/df). The fit of the model was judged based on the chi-square test of model fit and degrees of freedom (*χ*^2^/df < 5), RMSEA (RMSEA<0.1), CFI (CFI > 0.9), Tucker–Lewis index (TLI > 0.9), and SRMR (SRMR<0.05).

#### Reliability evaluation

Cronbach’s α coefficient was used to assess the internal consistency of the total scale and subscales (50). This method involves calculating the Cronbach’s α coefficients of the scale. An acceptable internal consistency is ensured with a coefficient greater than 0.7 (51).

#### Data collection

The two databases were collected through online platforms, and questionnaire completion was voluntary and anonymous. The first version of the HOES (22 items) was administered to a sample of 447 clinicians and nurses in Beijing Hospital Y, a tertiary hospital, from May 13 to May 20, 2021. Similarly, the final version of the HOES (18 items) was administered to a sample of 424 participants from Beijing Hospital S, a tertiary hospital, from June 10 to July 19, 2021. The valid response rate of the questionnaire was 76.8%.

## Results

### Preliminary item pool

According to the initially constructed conceptual framework, after referring to existing scales and published literature, a 32-item questionnaire was drafted, including the hospital culture (14 items), work situation (9 items), organizational support (5 items), and doctor–patient relationships (4 items) dimensions.

To make the organizational environment concept more in accordance with the actual work environment and occupational characteristics of Chinese medical staff, this study conducted semi-structured interviews to supplement the item pool. The interview results showed that the elements of the hospital organizational environment were basically consistent with the preliminary framework of the scale, except that scientific research situation was found to be important in public hospitals. Hence, the item pool comprised 36 items and the following 5 dimensions: hospital culture (14 items), work situation (9 items), organizational support (5 items), doctor–patient relationships (4 items) and scientific research situation (4 items).

### Content validity

The experts discussed the initial scale items repeatedly by using the focus group discussion method, deleting items with similar and irrelevant expressions, and adjusting the order and wording of sentences to form the initial scale. Any similar or ambiguous items were grouped together or excluded after two rounds of expert meetings. The development of the scale strictly followed the scientific scale preparation process and integrated the theories related to organizational environments to ensure the systematic and comprehensive nature of this scale. Interviews verified the adaptability of the scale dimensions and items in the current era, and the questionnaire items were basically compiled based on the mainstream scale items. Hence, the content validity of all items was proven to be appropriate, comprehensive, clear and understandable. The number of initial items was reduced to 22.

### Demographic data of participants

In this study, a total of 424 medical staff members were recruited from Hospital S, and 447 medical staff members were recruited from Hospital Y. Table 1 shows that the sex gap of the hospital’s medical staff was wide, with more women than men.

**Table 1:** Demographic characteristics of the participants.

Variable		Hospital S *N* = 424 (%)	Hospital Y *N* = 447 (%)
Age (years)	≤30	107 (25.2)	75 (16.7)
	31–40	236 (55.7)	211 (47.2)
	41–50	58 (13.7)	112 (25.1)
	≥51	23 (5.4)	49 (10.7)
Sex	Male	64 (15.1)	57(12.8)
	Female	360 (84.9)	390(87.2)
Marital status	Unmarried	77 (18.2)	71(15.9)
	Married	340 (80.2)	365(81.7)
	Separated/divorced	7 (1.6)	11(2.5)
Professional title	Primary title and below	252 (59.4)	163 (36.5)
	Middle title	122 (28.8)	197 (44.1)
	Vice-senior title	38 (9.0)	51 (11.4)
	Senior title	12 (2.8)	36 (8.1)
Human resources	Officially enrolled	226 (53.3)	297 (66.4)
	Officially unenrolled	194 (45.8)	147 (32.9)
	Other situations	4 (9)	3 (7)
Medical work experience (years)	<5	105 (24.8)	46 (10.3)
	6–10	133 (31.4)	104 (23.3)
	11–20	114 (26.9)	171 (38.3)
	21–30	58 (13.7)	85 (19.0)
	30	14 (3.3)	41 (9.2)

The medical staff were mainly concentrated in group aged 31–40 years old, which was the main working age. A large number of medical staff in the two hospitals were married and had a primary title; for the most part, the medical staff were officially enrolled and did not have a large number of working years.

### Descriptive analysis of items

As shown in Table 2, the average item value was 2.62–4.47, the standard deviation was 0.59–1.35, the floor effect (score = 1) was 0.23–23.11%, and the ceiling effect (score = 5) was 0.94–57.34%. In this study, there was almost no floor effect in the hospital organizational environment questionnaire for medical staff, but there was a ceiling effect, especially in the hospital culture dimension. Although there was ceiling effect, the proportion of participants with the lowest score and the highest score at the dimension level was less than 15%. It can be considered that there was no floor or ceiling effect at the dimension level. In addition, the skewness coefficient of each item was between −1.616 and 0.285, and the kurtosis coefficient was between −0.983 and 2.551. The data can be regarded as having an approximately normal distribution.

**Table 2:** Descriptive analysis of initial questionnaire item scores.

Items	Mean ± SD	Skewness	Kurtosis	Floor effect (%)	Ceiling effect (*%*)
Item 1	4.47 ± 0.788	−1.616	2.551	0.45	53.30
Item 2	4.62 ± 0.586	−1.357	1.170	0.46	57.34
Item 3	4.33 ± 0.854	−1.278	1.338	0.67	53.02
Item 4	4.48 ± 0.742	−1.546	2.432	0.23	0.94
Item 5	4.38 ± 0.823	−1.496	2.515	1.13	1.12
Item 6	4.45 ± 0.764	−1.388	1.662	0.56	58.83
Item 7	4.40 ± 0.783	−1.253	1.230	0.54	50.70
Item 8	3.89 ± 1.115	−0.904	0.126	4.53	33.25
Item 9	4.18 ± 0.961	−1.188	1.037	1.85	46.31
Item 10	2.77 ± 1.349	0.194	−1.156	22.82	13.42
Item 11	3.05 ± 1.329	−0.098	−1.129	13.44	16.55
Item 12	4.14 ± 0.853	−0.778	0.154	1.65	39.82
Item 13	2.79 ± 1.305	0.221	−1.054	14.15	12.98
Item 14	3.81 ± 1.032	−0.634	−0.285	2.01	28.86
Item 15	2.75 ± 1.284	0.285	−0.983	15.33	12.53
Item 16	4.23 ± 0.857	−1.063	0.993	0.94	38.68
Item 17	4.19 ± 0.871	−0.906	0.495	2.34	37.03
Item 18	4.30 ± 0.799	−1.080	1.028	10.37	43.86
Item 19	4.13 ± 0.909	−0.781	0.035	11.86	35.38
Item 20	2.62 ± 1.268	0.219	−0.879	23.11	10.07
Item 21	2.85 ± 1.299	0.126	−1.009	19.46	13.87
Item 22	3.19 ± 1.290	−0.215	−0.983	13.65	16.98

### Primary evaluation

#### Discriminant analysis

The discriminant analysis results showed that all items were significant in the high and low groups (*p* < 0.001) (Table 3). In this stage, the 22-item version appeared to have discrimination and to warrant further development.

**Table 3:** HOES item analysis.

Item	CR	Corrected item-total correlation	Cronbach α if the item is deleted
1. The hospital has a harmonious working atmosphere and a good culture	−18.037^***^	0.690^**^	0.902
2. Colleagues get along well and help each other	−16.311^***^	0.637^**^	0.904
3. The smooth coordination between hospital departments can effectively solve problems for patients	−19.556^***^	0.725^**^	0.901
4. Leaders have strong leadership and decision-making abilities	−17.743^***^	0.715^**^	0.902
5. Hospital functional departments have strong executive abilities	−17.417^***^	0.706^**^	0.901
6. The hospital provides a good opportunity for my promotion to a professional title	−20.709^***^	0.694^**^	0.902
7. The hospital does its best to provide me with training and exchange opportunities	−17.980^***^	0.736^**^	0.901
8. I’m satisfied with my salary and performance awards	−19.249^***^	0.645^**^	0.902
9. The working environment of the hospital is clean and comfortable	−19.539^***^	0.696^**^	0.901
10. I always have work to do	−9.003^***^	0.505^**^	0.908
11. I often work overtime in my job	−8.245^***^	0.533^**^	0.907
12. I can handle the current clinical work stress	−9.125^***^	0.536^**^	0.905
13. There are occupational exposures around me that could endanger my health	−7.080^***^	0.459^**^	0.909
14. Patients are courteous and respectful during the provision of medical care	−7.603^***^	0.472^**^	0.907
15. I have occasionally received verbal or violent threats or injuries from patients in my work	−5.808^***^	0.373^**^	0.911
16. The hospital respects my goals and values	−20.048^***^	0.747^**^	0.900
17. When I need special help, the hospital will help as much as possible	−18.556^***^	0.719^**^	0.901
18. The hospital cares about the health of the staff	−15.852^***^	0.701^**^	0.902
19. My opinions and suggestions on hospital development are listened to	−22.208^***^	0.742^**^	0.900
20. I feel much pressure from my research work	−11.656^***^	0.497^**^	0.907
21. I’m worried about how to complete research tasks	−12.726^***^	0.540^**^	0.906
22. I’m depressed and unhappy about my scientific work	−15.908^***^	0.557^**^	0.906

**p < 0.01, ***p < 0.001; CR (critical ratio).

#### Correlation analysis

According to the results of the overall correlation analysis (Table 3), the correlation coefficients between each variable and the total score of the 22 items were statistically significant (*p* < 0.01), and the absolute value and the new dimension score of each variable were the highest, indicating that each variable had good representativeness in its dimension. In this stage, the 22-item version of the questionnaire appeared to have sufficient correlation and to warrant further development.

#### Homogeneity analysis

The homogeneity analysis results are shown in Table 3. Items 13 and 15 were removed due to the risk of reducing the overall reliability of the scale. Twenty items were retained in the scale after the item analysis.

### Construct validity

#### EFA

An EFA was performed on the data obtained from 447 medical staff in Hospital Y, which initially generated five factors (KMO = 0.927, Bartlett’s test of sphericity *χ*^2^ = 7767.003, df = 190, *p* < 0.001) with a total explained variance of 71.210%. However, the fit was poor, and one factor was removed, as the eigenvalue of 0.471 was lower than 1. Two items (Items 12 and 14) were omitted due to nonsignificant factor loadings (< 0.5). After the final round of EFA (KMO = 0.921, Bartlett’s test of sphericity *χ*^2^ = 7394.295, df = 153, *p* < 0.001) on the remaining 18 items, 4 factors were produced, explaining 78.606% of the variance, and each eigenvalue was over 1 (Table 4). The explained variance of these four factors was 33.829%, 20.604%, 14.956%, and 9.217%, respectively. Based on the EFA results, the first factor contained nine items and was identified as hospital culture. The second factor was defined as work situation and contained two items. Four items were loaded on the third factor, which was defined as organizational support. Finally, three items described the scientific research situation. Overall, in the primary evaluation, most items fell into the corresponding dimensions, so it could be preliminarily stated that the HOES had good structural validity.

**Table 4:** Factors extracted from the HOES.

Items	Factor 1	Factor 2	Factor 3	Factor 4	Eigenvalue	% of variance
**Hospital Culture (HC)**						
1. The hospital has a harmonious working atmosphere and a good culture	**0.806**	0.291	0.094	−0.005	6.089	33.829
2. Colleagues get along well and help each other	**0.794**	0.192	0.114	−0.010		
3. The smooth coordination between hospital departments can effectively solve problems for patients	**0.820**	0.264	0.084	0.100		
4. Leaders have strong leadership and decision-making abilities	**0.855**	0.295	0.049	0.034		
5. Hospital functional departments have strong executive abilities	**0.829**	0.313	−0.002	0.101		
6. The hospital provides a good opportunity for my promotion to a professional title	**0.747**	0.379	0.015	0.093		
7. The hospital does its best to provide me with training and exchange opportunities	**0.757**	0.458	0.038	0.049		
8. I’m satisfied with my salary and performance awards	**0.545**	0.497	−0.033	0.117		
9. The working environment of the hospital is clean and comfortable	**0.672**	0.417	0.040	0.100		
**Work Situation (WS)**					1.659	9.217
10. I always have work to do	0.062	0.047	0.280	**0.877**		
11. I often work overtime in my job	0.117	0.035	0.343	**0.837**		
**Organizational Support (OS)**					3.709	20.604
16. The hospital respects my goals and values	0.476	**0.775**	0.079	0.053		
17. When I need special help, the hospital will help as much as possible	0.408	**0.832**	0.072	0.031		
18. The hospital cares about the health of the staff	0.439	**0.791**	0.095	−0.039		
19. My opinions and suggestions on hospital development are listened to	0.475	**0.789**	0.055	0.060		
**Scientific Research Situation (SRS)**						
20. I feel much pressure from my research work	0.025	0.031	**0.872**	0.250	2.692	14.956
21. I’m worried about how to complete research tasks	0.072	0.032	**0.929**	0.193		
22. I’m depressed and unhappy about my scientific work	0.080	0.104	**0.902**	0.157		

The bold values indicate the factor loading coefficients for the item under that dimension.

#### CFA

**Figure 2:**
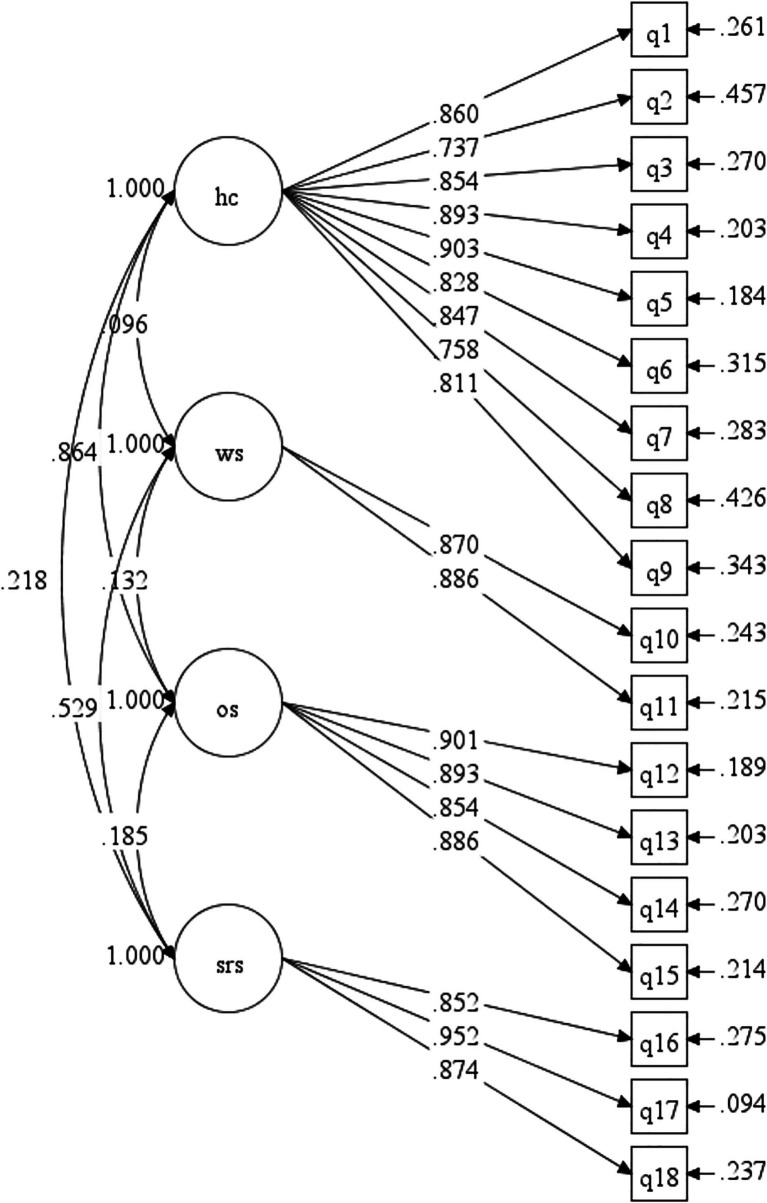
The CFA model of the HOES. HC, hospital culture; WS, work situation; OS, organizational support; SRS, scientific research situation.

The extracted factor structure was evaluated using CFA, and data were obtained from the 424 participants in Hospital S. The goodness-of-fit of the four-factor structure model (Figure 2) of the HOES was determined. The calculated goodness-of-fit indices were as follows: *χ*^2^/df = 3.273, CFI = 0.957, RMSEA = 0.074, TLI = 0.949, and SRMR = 0.035. These indices confirmed the model’s goodness-of-fit.

#### Convergent and discriminant validity

As shown in Table 5, the AVE was greater than 0.5 for all factors, and the CR value was greater than the AVE, which indicated great convergent validity. In addition, the AVE of Factors 2, 3, and 4 was greater than the MSV, and the ASV of four factors was less than the AVE. The discriminant validity of the HOES was confirmed.

**Table 5:** Convergent validity, discriminant validity, and reliability indices of the HOES.

Index Factor	AVE	MSV	ASV	CR	Cronbach’s alpha
Hospital Culture (HC)	0.697	0.744	0.375	0.954	0.935
Work Situation (WS)	0.776	0.303	0.240	0.874	0.824
Organizational Support (OS)	0.785	0.744	0.370	0.936	0.943
Scientific Research Situation (SRS)	0.804	0.303	0.298	0.925	0.920

AVE, Average Variance Extracted; MSV, Maximum Shared Squared Variance; ASV, Average of squared variance; CR, Composite reliability.

#### Reliability

As shown in Table 5, the Cronbach’s alpha coefficient (α) for the total scale was 0.910, which is considerably higher than the recommended value of 0.70. The internal consistency and composite reliability indices of the four dimensions were greater than 0.7, confirming the acceptable internal consistency and reliability of the factors. The scale reflecting the hospital organizational environment ultimately included four dimensions and 18 items.

#### Scoring

The final version of the HOES and the scores of medical staff in the two hospitals are shown in Table 6. We calculated the mean scores of the four dimensions by dividing the sum of the scores by the number of items. Then, we added the average scores of each dimension to obtain the score of the full scale. The total HOES score of the medical staff in Hospital S was 14.19 ± 2.75, while that of the medical staff in Hospital Y was 14.36 ± 2.77. There was no difference in the total scores or scores on the 4 dimensions between the two hospitals (*p* > 0.05). In addition, the total mean HOES score of the doctors was 14.18 ± 2.63, while that of the nurses was 14.36 ± 2.86. The univariate analysis results of this study showed that there were no significant differences in the total scores and scores on 3 dimensions between the doctors and nurses (*p* > 0.05). There was a significant difference in the scientific research situation score between the doctors and nurses (*p* < 0.05).

**Table 6:** The final version of the HOES and the scores of medical staff in the two hospitals.

Scale/items	Hospital S *N* = 424	Hospital Y *N* = 447	*T* test *P*	Doctors *N* = 392	Nurses *N* = 479	*T* test *P*
	$\bar{x}$ ±SD	$\bar{x}$ ±SD		$\bar{x}$ ±SD	$\bar{x}$ ±SD	
**Total scale**	14.19 ± 2.75	14.36 ± 2.77	>0.05	14.18 ± 2.63	14.36 ± 2.86	>0.05
**Hospital Culture (HC)**	4.26 ± 0.72	4.36 ± 0.69	>0.05	4.36 ± 0.68	4.27 ± 0.73	>0.05
1. The hospital has a harmonious working atmosphere and a good culture						
2. Colleagues get along well and help each other						
3. The smooth coordination between hospital departments can effectively solve problems for patients						
4. Leaders have strong leadership and decision-making abilities						
5. Hospital functional departments have strong executive abilities						
6. The hospital provides a good opportunity for my promotion to a professional title						
7. The hospital does its best to provide me with training and exchange opportunities						
8. I’m satisfied with my salary and performance awards						
9. The working environment of the hospital is clean and comfortable						
**Work Situation (WS)**	3.01 ± 1.23	2.91 ± 1.23	>0.05	2.87 ± 1.23	3.02 ± 1.23	>0.05
10. I always have work to do						
11. I often work overtime in my job						
**Organizational Support (OS)**	4.11 ± 0.81	4.21 ± 0.79	>0.05	4.21 ± 0.80	4.12 ± 0.79	>0.05
12. The hospital respects my goals and values						
13. When I need special help, the hospital will help as much as possible						
14. The hospital cares about the health of the staff						
15. My opinions and suggestions on hospital development are listened to						
**Scientific Research Situation (SRS)**	2.81 ± 1.20	2.86 ± 1.19	< 0.05	2.74 ± 1.16	2.93 ± 1.22	<0.05
16. I feel much pressure from my research work						
17. I’m worried about how to complete research tasks						
18. I’m depressed and unhappy about my scientific work						

## Discussion

In this study, the Chinese Hospital Organizational Environment Scale was developed through a standard and rigorous questionnaire development process. In the questionnaire development process, based on the actual work situation and psychological state of medical staff in Chinese public hospitals, a five-point Likert scale was used to enable medical staff to describe their current organizational environment more objectively. Based on our findings, the HOES had good internal consistency and validity. The acceptable explained variance of the scale confirmed its ability to measure the work environment among medical staff in China and could stably, reliably and accurately reflect the current level of the organizational environment perceived by Chinese medical staff.

This is the first study considering both clinicians and nurses to develop a detailed validation of a scale to assess hospital organizational environments. It applies to a wider subject group than previous scales targeting nurses. Compared to the Practice Environment Scale of the Nursing Work Index (NWI-PES) (22), the HOES developed in this study is a more specific tool that integrates factors related to practical conditions; the NWI-PES is composed of 31 items and 5 subscales: nurse participation in hospital affairs; nursing foundations for quality of care; nurse manager ability, leadership, and support of nurses; staffing and resource adequacy; and collegial nurse–physician relationships (52). Based on the actual situation in China, the constituent factors of the hospital organizational environment were summarized in this study. The HOES contains 18 items, five of which are reverse scored, and 4 subscales. Higher scores indicate a better work environment perceived by medical staff in China.

The first HOES subscale is hospital culture, which contains 9 items that refer to the overall hospital atmosphere. Hospitals with a “people-oriented” management culture realize the common value orientation of employees as the core, with the goal of developing team spirit (12). At present, China’s medical and health system reform has begun to improve the welfare of medical workers, focusing on their long-term career development. Only when the hospital culture is humanized can the organizational environment of the hospital be optimized and its development be sustainable.

The second HOES subscale is work situation, with 2 items reflecting the work intensity and work hours of the medical staff, which focus on the characteristics of the work itself and the occupational risks (24). Tertiary public hospitals are the main providers of medical services in China. Although a hierarchical medical system has been implemented, most patients still choose tertiary public hospitals for treatment when they first become ill due to the limited resources of primary medical and health institutions and inadequate medical service levels (53). This leads to a heavy workload and long work hours for medical staff in tertiary public hospitals, which is a problem that should be urgently addressed.

The third HOES subscale is organizational support, with 4 items evaluating the degree of support from the organization for the staff’s well-being (13). One study (54) showed that organizational support can affect doctors’ job satisfaction. The support and recognition perceived by hospital medical staff could generate positive work attitudes and enthusiasm. In contrast, if medical staff do not feel that their efforts and contributions are valued, their cognition can weaken their enthusiasm and sense of responsibility in the hospital and even lead them to consider leaving their jobs (55). Thus, hospital organizational support is a key part of the organizational environment that determines the working attitudes of medical staff.

The fourth HOES subscale is the scientific research situation, with 3 items representing the most defining characteristics of the Chinese hospital environment combined with the characteristics of the hospital performance appraisal, professional title promotion and other systems (56). In addition to their clinical practice, most medical staff have no choice but to refer to a large number of studies to prepare for scientific research because achievements in scientific research are related to their promotion and salary. Chinese medical staff are thus forced to carry out scientific research projects.

The correlation coefficients among the HOES dimensions were statistically significant. The contents of the scale had high representativeness, high internal consistency, and good reliability. The EFA and CFA results showed that the fit index of the scale was good, which indicated that the questionnaire had good validity. The physician–patient relationship dimension was deleted due to low reliability; the reason may be that this questionnaire survey was conducted after the COVID-19 outbreak. During the COVID-19 period, medical staff were rushed, which made patients more tolerant and more understanding of doctors and nurses. In addition, Chinese hospitals implemented stricter patient management, and only critical patients can choose to be treated in tertiary hospitals. As a result, the number of patients in tertiary public hospitals decreased during the pandemic, causing physician–patient relationships to improve. Ultimately, the internal consistency of the final version of the scale was 0.910 and ranged from 0.824 to 0.943 in each subdimension. There was no significant difference in the total score or the scores of the four dimensions between the two hospitals. This result indicated that the organizational environments of the two hospitals were similar. The reason may be that the two hospitals are tertiary hospitals with little difference in the service scale and overall volume of consultations and medical treatment; hence, the overall environments perceived by the medical staff were similar. The univariate analysis results of this study showed that nurses’ perceived organizational environment scores on the scientific research situation dimension were higher than those of doctors. Under the current professional title promotion system, if medical staff want to be rated at or above the intermediate title, they not only need to have enough years of work, but also need to complete daily rounds, host meetings on certain topics, publish enough papers, complete credit courses required for continuing medical education, and even complete tasks and work such as teaching and providing care in the countryside (57, 58). In the future, cooperation between doctors and nurses in scientific research can be strengthened to help hospitals provide a harmonious organizational environment. There is an urgent need for more evidence on scientific research situation in medical staff.

## Strengths and limitations

The strengths of the study are as follows: we developed and validated a scale by using a Chinese sample of medical staff for potential application in hospital management. This study provides a new tool from new perspectives that can be adopted among medical staff to assess the organizational environment in hospitals in China and other overseas regions with similar situations. The analysis of the perceived organizational environment provides protection for the physical and mental health of medical staff. This study serves as a foundation for developing the hospital organizational environment of clinicians and nurses to enhance hospital staff management.

There were several limitations in the current study. First, the samples in this study were selected from two tertiary hospitals (the mainstay of medical care in China) in Beijing and cannot represent secondary or primary hospitals in other regions. More HOES validation studies should be conducted to verify its suitability for different regions and different levels of hospitals in a wide area. In addition, we did not perform a comparison with the Nursing Work Evaluation Index due to the limitations of the current research site, so future studies should continue to explore the validity of the HOES and compare it with the Nursing Work Evaluation Index in a context in which the nurse population is large. Future studies should be conducted to explore the sustainability and stability of the results across such periods.

## Conclusion

The HOES is a comprehensive instrument with demonstrated validity and reliability that can be adopted among medical staff to assess the organizational environment in hospitals. The tool designed in this study was used to assess the organizational environments of clinicians and nurses. Since the scale was developed based on the Chinese context, more studies are needed to support the adaptation of the HOES in other contexts.

## Data availability statement

The datasets presented in this article are not readily available because data may be made available by contacting the corresponding author. Requests to access the datasets should be directed to KM. E-mail: mengkai@ccmu.edu.cn.

## Ethics statement

The studies involving humans were approved by the Ethical Review Committee of Capital Medical University (No. Z2021SY011). The studies were conducted in accordance with the local legislation and institutional requirements. The participants provided their written informed consent to participate in this study.

## Author contributions

YW and JZ contributed to the data curation, software analysis, formal analysis, and writing of the original draft and are responsible for the overall content as the guarantors. XF and YL participated in the study investigation and validation, and made recommendations for the manuscript draft. KM and ZG contributed to the conceptualization, methodology development, writing, and review and editing of the manuscript, project supervision and administration, and funding acquisition. All authors contributed to the article and approved the submitted version and complied with the Human Resources for Health standards for authorship.

## Funding

This study was supported by the National Natural Science Foundation of China (72074160) and the Natural Science Foundation of Beijing (9222004). The funders had no role in any aspects of this study, including the study design, data collection and analysis, decision to publish or preparation of the manuscript.

## Conflict of interest

The authors declare that the research was conducted in the absence of any commercial or financial relationships that could be construed as a potential conflict of interest.

## Publisher’s note

All claims expressed in this article are solely those of the authors and do not necessarily represent those of their affiliated organizations, or those of the publisher, the editors and the reviewers. Any product that may be evaluated in this article, or claim that may be made by its manufacturer, is not guaranteed or endorsed by the publisher.
